# Benign esophageal schwannoma: A case report and brief overview

**DOI:** 10.1097/MD.0000000000021527

**Published:** 2020-07-31

**Authors:** Chun-Xiao Wu, Qi-Quan Yu, Wei-Zhen Shou, Kun Zhang, Ze-Quan Zhang, Qi Bao

**Affiliations:** a Department of Thoracic Surgery, Longhua Hospital, Shanghai University of Traditional Chinese Medicine, Xuhui District; b Department of Thoracic Surgery, Ruijin Hospital, Shanghai Jiaotong University School of Medicine, Shanghai, China.

**Keywords:** esophageal schwannoma, histopathology, image, surgery, symptom

## Abstract

**Rationale:**

Schwannoma is a tumor of the peripheral nervous system that originated in the Schwann cells of the neural sheath. Esophageal schwannomas are rare esophageal submucosal tumors, comprising approximately 2% of esophageal tumors. Since the symptoms, signs, and images of esophageal schwannoma are not specific, its preoperative diagnosis remains challenging.

**Patient concerns:**

A 67-year-old woman visited our department with complaints of gradually developed dysphagia and dyspnea for 4 years. A chest computed tomography scan showed a well-demarcated, enhancing homogeneous tumor measuring 61 × 46 × 60 mm in the upper third of the esophagus. Upper gastrointestinal endoscopy revealed a smooth elevated lesion located 19 to 24 cm from the incisor teeth. An endoscopic ultrasound-guided fine-needle aspiration demonstrated the presence of benign spindle cells.

**Diagnoses:**

Histopathologic examination revealed spindle-shaped cells in a fasciculated and disarrayed architecture. The immunohistochemical study showed positivity for S-100 protein antibody and absence of staining for CD117, CD34, smooth muscle actin, and Desmin. These findings confirmed the diagnosis of benign esophageal schwannoma.

**Interventions:**

The tumor was considered to be difficult to repair the esophagus by direct anastomosis after tumor resection. Therefore, subtotal esophagectomy and esophagogastrostomy in the right thorax were performed.

**Outcomes:**

The patient has been doing well with no recurrence at 36 months after the operation.

**Lessons:**

The symptoms and surgical procedures for benign esophageal schwannoma depend on the size and location of the tumor, proper and timely treatment is essential. A definitive diagnosis is confirmed by histology, and complete excision should yield good results.

## 1 Introduction

Schwannoma or neurilemmoma is a tumor of the peripheral nervous system originated in Schwann cells, and it usually refers to a benign, slow-growing tumor.^[1]^ This type of tumor occurs more frequently in the head and neck, extremities, and retroperitoneum. Rarely, it can be found in the gastrointestinal tract and esophagus.^[2]^ Esophageal schwannomas are sporadic and are the least common esophageal submucosal tumors (SMTs), and account for approximately 2% of all esophageal tumors. They are commonly located in the upper and mid-esophagus in the mediastinum. The mean age of patients is 50 years, with a female predominance,^[3]^ there is a strong predominance for this disease entity in the Asian population.^[4]^ As there is no characteristic manifestation in the image of esophageal schwannoma, preoperative diagnosis of schwannoma is regarded to be difficult,^[3]^ and the definitive diagnosis is often established after resection.^[5]^ Therefore, misdiagnosis usually occurs. In the following study, we report a patient with esophageal schwannoma. In this case report, we describe our surgical experience with benign esophageal schwannomas, with a brief review of other reported cases in the literature.

## 2 Case report

**Figure 1:**
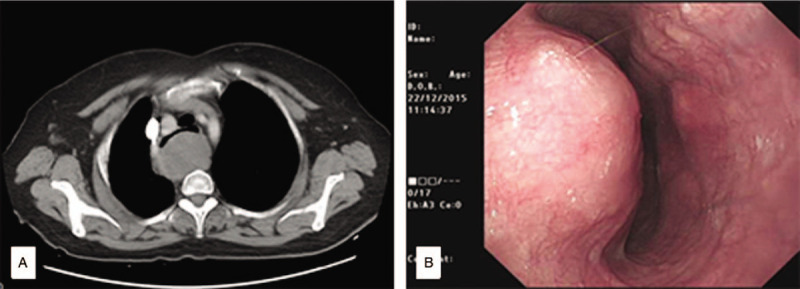
The preoperative radiologic findings. Computed tomography (CT) scan (A) and endoscopy (B) showing the tumor. (A) A CT scan showing a large mass (61 × 46 × 60 mm) in the upper third of the esophagus. (B) Esophagogastroduodenoscopy showed a smooth elevated lesion located 24 to 30 cm from the incisor teeth, the mucosa was intact.

A 67-year-old Chinese woman presented for evaluation of difficulty swallowing and dyspnea. She had been aware of dysphagia in the past 1 year. A computed tomography (CT) scan showed a well-demarcated, enhancing homogeneous tumor measuring 45 × 45 × 33 mm in the upper third of the esophagus. However, since this symptom seemed mild, she rejected further evaluation and treatment. The patient came back after 3 years because the symptoms of swallowing and dyspnea were progressively aggravated. The physical examination showed no significant findings, and laboratory tests including serum tumor markers such as CEA and CA19-9 were normal. At this time, the CT scan revealed a posterior mediastinal mass, about 61 × 46 × 60 mm in size (Fig. 1A). The tumor showed a heterogeneous pattern and strongly compressed the esophagus and trachea, no regional lymph node enlargement was observed. Esophagogastroduodenoscopy showed a smooth elevated lesion located 24 to 30 cm from the incisor teeth, and the mucosa was intact (Fig. 1B). Endoscopic ultrasonography guided fine-needle aspiration (EUS-FNA)-mediated histopathologic examination revealed a proliferation of spindle-shaped cells in a fasciculated and disarrayed architecture. Because the specimen was small, we were unable to establish a definitive diagnosis. We suspected an esophageal schwannoma, leiomyoma, or gastrointestinal stromal tumor. The patient underwent surgery because of her difficulty swallowing and our suspicion of malignant potential.

The patient was placed in the left lateral position and underwent anterolateral thoracotomy via the 4th right intercostal space. A mass about 10 × 5 cm in size was found adjacent to the upper and thoracic esophagus. Although preservation of the mucosal layer with tumor enucleation is a less invasive technique, in our case, full-thickness excision was indicated because of dense adhesions between the tumor and the surrounding esophagus as well as thinning of the mucosal and muscular layers. A total thoracic esophagectomy with cervical esophagogastrostomy, pyloroplasty, and feeding jejunostomy was performed. The postoperative course was uneventful. The patient was discharged on the 12th postoperative day. The patient has been doing well with no recurrence at 36 months after the operation.

**Figure 2:**
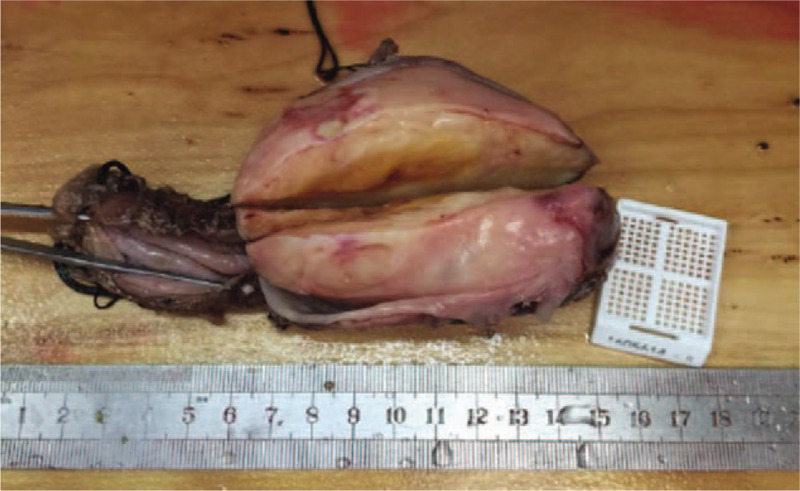
The resected specimen showing a well-demarcated and elastic hard appearance in the submucosal layer and was measured 90 × 60 × 45 mm, the cut surface was almost uniformly pale yellow.

**Figure 3:**
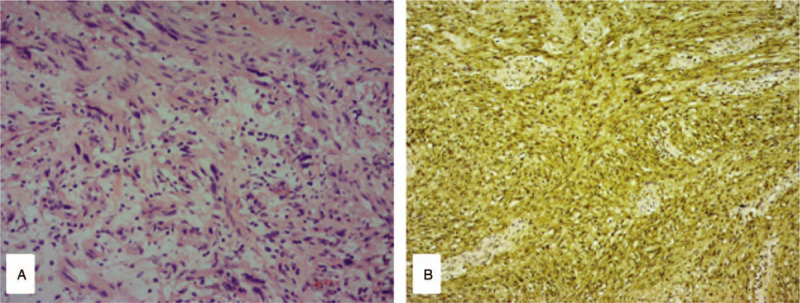
Histology and immunohistochemistry of the tumor. (A) Histopathologic findings revealed spindle-shaped cells in a fasciculated and disarrayed architecture, a lymphoid cuff was also detected, and no pathologic mitosis was observed (hematoxylin and eosin staining; magnification, ×200). (B) S100 protein staining (magnification, ×200).

The specimen showed a well-demarcated and elastic hard appearance and was measured 90 × 60 × 45 mm, the cut surface was almost uniformly pale yellow (Fig. 2). Hematoxylin and eosin staining revealed spindle-shaped cells in a fasciculated and disarrayed architecture, a lymphoid cuff was also detected, and no pathologic mitosis was observed (Fig. 3A). Immunohistochemical examination revealed S-100 protein positivity (Fig. 3B) and CD117, CD34, DOG-1, Ki-67, SMA, Syn, NSE, and P53 negativity, establishing the diagnosis of esophageal schwannoma.

The patient has authorized and provided informed consent for the publication of her medical data. The present study was approved by the Ethics Committees of the Longhua Hospital, Shanghai University of Traditional Chinese Medicine, and the Ethics Committees of the Ruijin Hospital, Shanghai Jiaotong University School of Medicine.

## 3 Discussion

Esophageal schwannoma was first described by Chatelin and Fissore in 1967.^[6]^ The first report in the English literature was by Eberlein and colleagues in 1992,^[7]^ reports about it are recently increasing. There have been only 28 reported cases in China in the literature.^[8]^ Therefore, it is generally poorly recognized in the clinic and easy to be misdiagnosed before surgery. In the present case, we did not have a definitive preoperative diagnosis of esophageal schwannoma.

The symptoms of this disease depend on the location, patient with esophageal schwannoma complain a variety of symptoms including dysphagia, dyspnea, chest pain, and coughing,^[4]^ abdominal pain, constipation, bleeding, and weight loss could be manifested in the minority of cases, even no symptom until the tumors grow larger. The symptomatic analysis showed that the most frequent symptoms were dysphagia (53.7%), dyspnea (10.4%), cough (4.4%), weight loss (4.4%), and chest pain (4.4%).^[9]^ Only 1 case reported the tracheal obstruction from a benign esophageal schwannoma till now.^[10]^ Dysphagia is the most common symptom. The esophageal lumen is gradually narrow as the lesion increasing, but the food can pass through the lumen since the esophageal mucosa itself is not damaged, the process of swallowing is hindered by the lesion. The tolerable swallowing despite a large esophageal mass was possibly due to the tumor's characteristic of indolent growth and elasticity of the esophagus. Dyspnea is due to the adjacent relationship between the esophagus and the trachea in the superior mediastinum, the lesion in the upper esophagus can compress the trachea and cause difficulty in breathing. Chest pain and cough are referred to nerve reflections caused by lesions.

There is no apparent abnormality in the laboratory tests including serum tumor markers about esophageal schwannomas. Imaging examinations usually include X-ray, CT scan, magnetic resonance imaging, positron emission tomography (PET), EUS, esophagography, and endoscopy, but it is often difficult to confirm the diagnosis by imaging. A chest X-ray commonly reveals a widened mediastinum and esophageal deviation when the lesion is larger.^[11]^ CT scan is subsequent radiologic examination after X-ray, it demonstrates an upper esophageal mass that adhered to the posterior wall of the esophagus, the narrow trachea is seen to be bowing anteriorly caused by the compression. No necrosis or hemorrhage was evident, with homogeneous enhancement following injection of contrast. Magnetic resonance imaging of the chest revealed that the boundary of the tumor was clear and smooth. The mass exhibited iso-intensity or low intensity compared with the muscles on T1-weighted images and high intensity on T2-weighted images. There was no invasion into the surrounding tissue. [18F]-fluorodeoxyglucose (FDG)-PET CT showed a hypermetabolic appearance matching the tumor mass, the reason is that schwannomas originate from nerve cells that express glucose transporter type 3, and FDG uptake is considered to be increased by glucose transporter type 3. However, there was no correlation between FDG uptake and the proliferation rate (Ki-67 index). Therefore, FDG-PET could not reveal whether the hypermetabolic appearance indicated a benign or malignant tumor.^[12]^ EUS demonstrates a large tumor mass in the esophageal wall that is hypoechoic and appeared to originate from either the submucosal or the muscular layer. Esophagogastroduodenoscopy reveals a smooth elevated lesion at a position of 19 to 30 cm from the incisor teeth, and the mucosa is usually intact. The barium swallow demonstrates a smooth rounded indentation of the proximal esophageal lumen which does not impede the passage of orally administered barium.

Esophageal schwannoma is challenging to diagnose by standard imaging techniques; confirmation of the diagnosis requires pathologic examination with further immunostaining studies after surgical resection. Preoperative EUS-FNA may be useful for both the diagnosis and management of this disease.^[13]^ Rong et al reported that the diagnostic accuracy of EUS-FNAB for SMTs was 85.2%.^[14]^ However, there have been no reports of diagnosis of an esophageal schwannoma before surgery, because diagnosis by biopsy is difficult owing to a shortage of tissue and the submucosal benign tumor morphology. EUS-FNA may also have several procedural risks including bleeding and infection, although these risks are minimal.^[15]^ In general, histologic features of schwannoma include spindle-shaped tumor cells arranged in a palisading pattern or with loose cellularity in a reticular array.^[16]^ Most cases show 2 distinct histologic patterns, referred to as Antoni A and Antoni B.^[17,18]^ In Antoni A tissue pattern, the cells are spindle-shaped and compactly arranged. The pattern is characterized by palisades created by the alignment of nuclei that alternate with anucleated, rosy and homogenous or fibrillary zones (Verocay bodies). These bodies consist of cytoplasmic extensions, basement membranes, collagen and reticulin or small groups of fibrils surrounded by lines of palisade-shaped nuclei. In Antoni B tissue pattern, the cells are loosely arranged. The extensions are not oriented and the nuclei are round, rather to elongated. Occasionally, the cells are starred, creating a resemblance to an astrocytoma. There may be abundant xanthomatous histiocytes. The immunohistochemical and structural features of Antoni B regions suggest that it results from degenerative processes. The histologic criteria for the diagnosis of malignant schwannoma are based on a combination of the presence of mitotic figures, nuclear atypia, cellularity, and tumor necrosis, and these characteristics may be useful in distinguishing malignant schwannomas from benign.^[2]^ The identification of a neurogenic tumor cell origin is by positive immunostaining for S-100 and vimentin (1–3, 5–8), the characteristic markers of Schwann cells. Markers such as CD117 and CD34 will be negative, differentiating it from gastrointestinal stromal tumors. Smooth muscle cell markers, actin and desmin, will also be negative, differentiating it from leiomyomas.

The therapeutic management of esophageal schwannoma depends on several factors, such as clinical complaints, tumor size, complications due to tumor growth, and pathologic data (malignancy, mitotic index, and immunohistochemical staining).^[19]^ Chemotherapy and radiation therapy are ineffective, and the most common treatment is enucleation by surgery or endoscopy.^[3]^ Surgical treatment includes enucleation and esophagectomy with esophagogastrostomy; the surgical method includes thoracoscopic enucleation, robot-assisted thoracoscopic resection, and thoracotomy. The use of enucleation with video-assisted thoracoscopic surgery is becoming common for small tumors (≤2 cm).^[20]^ Although no specific cutoff for size could be identified, most tumors >7 cm were removed by thoracotomy.^[21]^ Thoracotomy or esophagectomy would be usually chosen for tumors that are 50 mm or more in size or located in the upper and middle 3rds of the thoracic esophagus, enucleation may not be a preferred approach for very large tumors because this has been associated with higher rates of esophageal stenosis.^[22]^ Esophageal surgery, especially for esophagectomy, is usually performed through the right thoracic cavity. Although the left thoracic approach may provide direct access to a tumor on the left esophageal wall, the aortic arch and the trachea may restrict this access, especially for tumors located in the upper and middle thoracic esophagus. Lymph node dissection for esophageal SMTs is controversial. When an esophageal SMT is suspected to be malignant based on concerning preclinical or radiographic findings (such as local invasion or enlarged suspicious lymph nodes), lymph node dissection should be considered. Radical esophagectomy with regional lymph node dissection may also be needed in certain cases to minimize potential recurrence.

In conclusion, esophageal schwannoma is a rare entity but must be a consideration when faced with a submucosal esophageal mass. The symptom and surgical procedures depend on the size and location of the tumor; proper and timely treatments are essential. A definitive diagnosis is made by histology, and complete excision should yield good results. The overwhelming majority of patients will not require esophagectomy, but for large lesions suspected of diffuse esophageal involvement, patients should be prepared for esophagectomy and esophagogastrostomy.

## Author contributions

**Conceptualization:** Chun-Xiao Wu, Qi Bao.

**Data curation:** Qi-Quan Yu, Wei-Zhen Shou, Kun Zhang.

**Investigation:** Qi-Quan Yu, Kun Zhang, Ze-Quan Zhang.

**Supervision:** Chun-Xiao Wu, Qi Bao.

**Writing – original draft:** Chun-Xiao Wu, Wei-Zhen Shou, Qi Bao.

**Writing – review & editing:** Chun-Xiao Wu, Kun Zhang, Ze-Quan Zhang, Qi Bao.
